# Antithrombotic therapy in coronary artery disease patients with atrial fibrillation

**DOI:** 10.1186/s12872-020-01609-8

**Published:** 2020-07-06

**Authors:** Lili Wei, Enyong Su, Weili Liu, Wenlu Xing, Xinyun Liu, You Zhang, Shan Wang, Qianqian Cheng, Datun Qi, Chuanyu Gao

**Affiliations:** 1 Department of Cardiology, Zhengzhou University People’s Hospital, No.7 Weiwu road, Jinshui District, Zhengzhou, 450003 Henan China; 2 Department of Health Management, Henan Provincial People’s Hospital, No. 7 Weiwu Road, Jinshui District, Zhengzhou, 450003 Henan China; 3 Department of Cardiology, Huazhong Fuwai Hospital, No. 1 Fuwai Road, Zhengzhou, 451464 Henan China; 4 Department of Cardiology, Zhongshan Hospital, Fudan University, No. 180 Fenglin Road, Xuhui District, Shanghai, 200032 China

**Keywords:** Coronary artery disease, Acute coronary syndrome, Atrial fibrillation, Triple therapy, Oral anticoagulant, Double antiplatelet therapy, Dual therapy, Bleeding

## Abstract

**Background:**

Coronary artery disease (CAD) and atrial fibrillation (AF) frequently coexist in clinical practice, making it challenging for the treating physician to choose anticoagulation and antiplatelet therapies. The aim of this study was to investigate antithrombotic strategies and assess related adverse outcomes in stable coronary artery disease (SCAD) and acute coronary syndrome (ACS) patients with AF when the CHA_2_DS_2_-VASc score was ≥2.

**Methods:**

We performed a retrospective study and collected data from a computer-based patient record management system in Zhengzhou University People’s Hospital in China. In total, 2978 patients with a hospital discharge diagnosis of CAD and concomitant AF who met the inclusion criteria were enrolled from January 1, 2012 to December 31, 2016, and data from 2050 patients were finally analysed. The χ^2^ test was used to compare the incidences of clinical endpoints between the SCAD+AF group and the ACS + AF group. Multivariable Cox regression analysis was performed to identify independent predictive factors of adverse outcomes in both groups.

**Results:**

Oral anticoagulant (OAC) monotherapy was the most common antithrombotic therapy in SCAD+AF patients (49.55%), while double antiplatelet therapy (DAPT) was the most common treatment in ACS + AF patients (54.19%) at discharge. OAC monotherapy significantly increased and the use of single antiplatelet therapy (SAPT) decreased during follow-up (34 ± 13 months) when compared to their use at discharge in the SCAD+AF group (all *p* < 0.001). In the ACS + AF group, the proportion of patients using DAPT decreased notably, while the proportions of patients using SAPT and dual therapy (DT) combining OAC with SAPT increased significantly during follow-up (all *p* < 0.001) compared to the proportions at discharge. According to multivariable Cox regression analysis, age, hypertension and prior stroke were independent risk factors for ischaemic stroke in the SCAD+AF group and ACS + AF group (all *p* < 0.05). OAC was an independent protective factor for ischaemic stroke in both groups (all *p* < 0.05). Previous bleeding independently increased the risk of haemorrhage in both groups (all *p* < 0.01).

**Conclusions:**

In this study, the proportion of anticoagulant-antiplatelet combined therapy was low in ACS + AF patients with high stroke risk. In clinical practice, the awareness of anticoagulation needs to be strengthened regarding patients with CAD and AF.

## Background

Antithrombotic strategies include antiplatelet therapy (APT), which suppresses platelet function, and oral anticoagulant (OAC) therapy, which interferes with the signalling cascade of blood coagulation. APT is regarded as the cornerstone of the treatment of patients with stable coronary artery disease (SCAD) and of patients after acute coronary syndrome (ACS) [1]. OAC is closely associated with atrial fibrillation (AF), which exclusively promotes the development of thrombotic conditions in the left atrium and left atrial appendage and increases the risk of ischaemic stroke [2, 3]. Coronary artery disease (CAD) and AF are the primary heart diseases worldwide and are responsible for the high morbidity and mortality of cardiovascular and cerebrovascular diseases. By 2050, it is estimated that the number of AF patients in Asia will reach 72 million, which is more than double the combined numbers in Europe and the United States [4]. Thirty percent of AF patients have concomitant CAD, and up to 15% of patients with SCAD also have AF [5, 6]. The prevalence of AF in patients with ACS ranges from 10 to 21% and increases with severity of myocardial infarction (MI) and age [7]. Patients with CAD and AF have a high incidence of both ischaemic and haemorrhagic events, which frequently coexist in daily practice. The combination of AF and CAD is a general and complicated problem and makes it challenging for the treating physician to choose anticoagulation and antiplatelet therapies. In this setting, it is essential for the treating physician to determine the antithrombotic regimen with the desired benefit/risk ratio for specific patients. Current guidelines recommend short-term triple therapy (TT), consisting of OAC and double antiplatelet therapy (DAPT), although TT inevitably leads to a higher incidence of major bleeding [2, 3, 8, 9]. However, the management of many patients with CAD and AF in the real world has long relied on medical experience rather than guidelines and consensus.

A few factors affect the development of thromboembolic events among patients with AF, such as ischaemic stroke and systemic embolism, and these factors are integrated into the CHA_2_DS_2_-VASc score [10]. The CHA_2_DS_2_-VASc score is applied to assess the risk level of patients with AF and can subsequently provide guidance on the reasonable use of OAC. In this single-centre observational and retrospective study from Central China, we aimed to study antithrombotic therapies targeting patients with CHA_2_DS_2_-VASc scores ≥2 with SCAD and ACS concomitant with AF in clinical practice, to evaluate related adverse outcomes, and ultimately to provide evidence for the choice of antithrombotic strategy in the future.

## Methods

### Study design and population

This work was approved by the Ethics Committee of Zhengzhou University People’s Hospital [NO. 2016(40)] and was exempted from the requirement of informed consent. This was an observational retrospective study based on patients with a discharge diagnosis of CAD and concomitant AF. Data were collected from a computer-based patient record management system in Zhengzhou University People’s Hospital in Henan Province, China, from January 1, 2012 to December 31, 2016. The database covered the central region of China, spanning approximately 167,000 km^2^ with a population of 0.96 billion inhabitants (2018 population statistics), representing 6.9% of the population of China. All information used for data analysis in this study was anonymized.

Patients who met all the following criteria were eligible for inclusion: 1) age above 18 years; 2) AF recorded on electrocardiogram (ECG) or Holter monitor during hospitalization; 3) coronary arteriography (CAG) or coronary computed tomography angiography (CTA) showing at least one stenosis of the great coronary artery ≥50% or typical electrocardiographic changes and elevated myocardium biochemical markers.

The exclusion criteria were as follows: 1) atrial flutter; 2) rheumatic valve disease; 3) any severe condition that would limit life expectancy to less than 1 year; 4) contraindication to the use of OAC, aspirin, or P2Y12 platelet inhibitors (clopidogrel, prasugrel or ticagrelor); and 5) reversible AF (caused by surgery, mental stress, hyperthyroidism, alcohol and exhaustion).

Patients with a CHA_2_DS_2_-VASc score ≥ 2 in this study were finally divided into the SCAD+AF group and the ACS + AF group for further analysis.

### Data collection

We gathered clinical characteristics such as age, history of hypertension and bleeding, types of AF, cardiovascular drugs at discharge, choice of operative procedures for cardiovascular events and risk scores for predicting cardiovascular complications, including the CHA_2_DS_2_-VASc score (congestive heart failure, hypertension, age > 75 years, diabetes mellitus, prior stroke with transient ischaemic attack or thromboembolism, vascular disease, age 65 to 74 years and sex) and the HAS-BLED score (hypertension, abnormal renal or liver function, stroke, bleeding, labile international normalized ratio [INR], elderly status [age > 65 years], and drug or alcohol use).

### Follow-up and outcomes

All patients were followed up for at least 1 year. Information concerning antithrombotic management strategy and clinical outcomes was obtained via phone calls to patients with standardized questions and was recorded. Further information was obtained by reviewing hospital discharge reports relating to any other readmission during the follow-up period. All patients were followed up until death or the end of the study period (December 31, 2018). Outcomes included 1) ischaemic stroke, demonstrated by imaging; 2) bleeding, classified according to the Global Utilization of Streptokinase and Tissue Plasminogen Activator for Occluded Coronary Arteries (GUSTO) criteria [11, 12] as major bleeding, including intracranial haemorrhage or haemodynamic disorders or haemorrhage requiring intervention; moderate bleeding requiring blood transfusions but not resulting in haemodynamic disorders; and mild bleeding, not qualifying as major bleeding or moderate bleeding according to diagnostic standards; 3) MI, presenting the typical ECG changes and increased troponin or confirmed by CAG; 4) all-cause death, defined as death from any cause; and 5) thromboembolism, including ischaemic stroke, MI and systemic embolic events.

### Definitions

ACS consists of unstable angina, ST-segment elevation myocardial infarction (STEMI), and non-ST-segment elevation myocardial infarction (NSTEMI) [12]. The assessment of SCAD referred to at least 12 months after the last coronary event with documented coronary stenosis by CAG or CTA [12, 13]. AF was diagnosed based on an ECG or Holter recording, and rheumatic valve AF and atrial flutter were excluded [14]. We defined the following antithrombotic regimens: single antiplatelet therapy (SAPT: aspirin or clopidogrel or ticagrelor), OAC (warfarin or rivaroxaban or dabigatran), DAPT (aspirin plus clopidogrel or ticagrelor), dual therapy (DT) referring to OAC plus SAPT, and TT referring to warfarin plus DAPT (aspirin plus clopidogrel).

### Statistical analysis

Continuous variables and categorical variables are described as the mean ± standard deviation and percentages, respectively. Continuous variables were compared using t-tests, while categorical variables were compared using χ^2^ tests. Multivariable Cox regression analysis was performed to identify independent predictive factors of adverse clinical events. A *p*-value < 0.05 was regarded as statistically significant. SPSS software (version 20.0) was used for performing calculations.

## Results

### Baseline characteristics

**Fig. 1:**
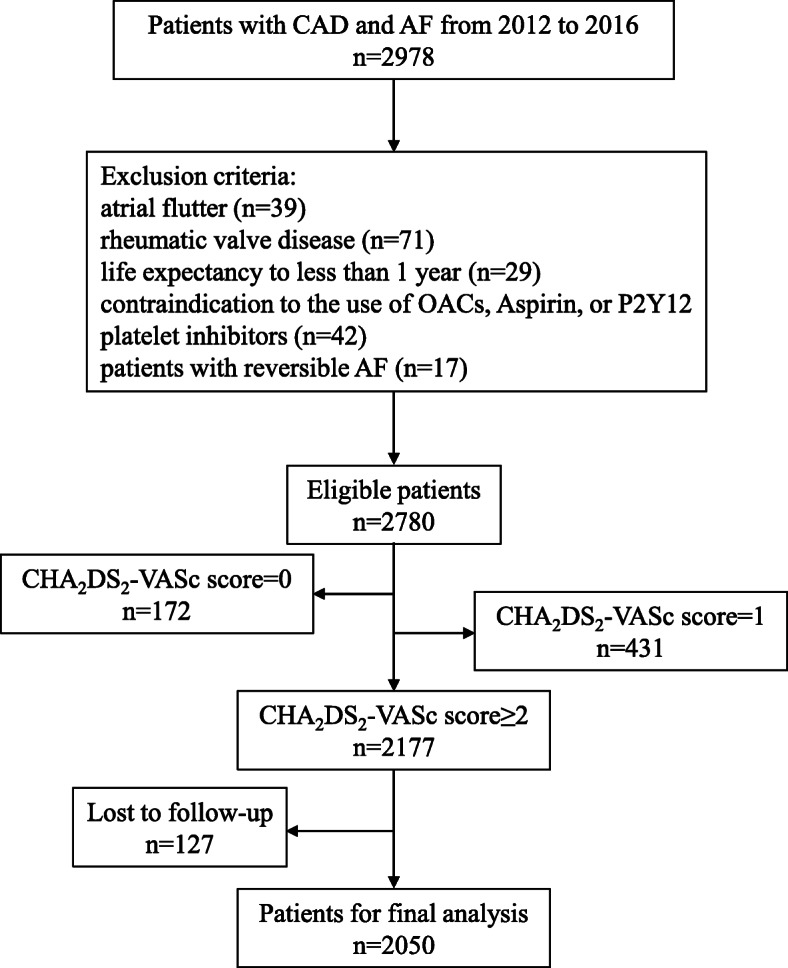
Study flow chart. Abbreviations: SCAD: stable coronary artery disease; ACS: acute coronary syndrome; AF: atrial fibrillation; OAC: oral anticoagulant

A total of 2978 patients with CAD and AF were enrolled from January 1, 2012 to December 31, 2016. A total of 198 patients were excluded, and 2780 patients were eligible. A total of 2177 patients had a CHA_2_DS_2_-VASc score ≥ 2, and 127 were lost to follow-up. Therefore, the final analysis included 2050 patients (Fig. 1). The characteristics of the study population at baseline are displayed in Table 1. There were 559 patients in the SCAD+AF group and 1491 patients in the ACS + AF group. Compared to the SCAD+AF group, the ACS + AF group showed a higher rate of percutaneous coronary intervention (PCI) (7.51% vs. 11.13%, *p* = 0.016). No significant difference was found in demographics, past medical history, cardiovascular drug therapies, types of AF, thromboembolism or bleeding risk scores between these two groups (all *p*>0.05). Statins were the most common medication prescribed and administered according to the patients’ records at discharge, both in the SCAD+AF group and ACS + AF group (88.55 and 86.38%, respectively). Hypertension was the most common complication in both groups (69.41 and 71.63%, respectively). Most patients who took warfarin for a long time exhibited INR controlled at 2–3 when they were discharged (Supplement Table 1).

**Table 1:** Baseline characteristics of study population

Characteristics	SCAD + AF (***n*** = 559)	ACS + AF (***n*** = 1491)	***P***-value
Female, n (%)	256 (45.80)	672 (45.07)	0.064
Age, (years)	71.12 ± 0.86	72.34 ± 0.76	0.841
Smoking, n (%)	172 (30.77)	450 (30.18)	0.796
Alcohol drinking history, n (%)	144 (25.76)	363(24.35)	0.509
ethanol per person per day, (grams)	5.12 ± 0.75	4.86 ± 0.33	0.712
Heart failure, n (%)	62 (11.09)	193 (12.94)	0.258
Arterial hypertension, n (%)	388 (69.41)	1068 (71.63)	0.324
Diabetes mellitus, n (%)	99 (17.71)	318 (21.33)	0.070
Hypercholesterolemia, n (%)	171 (30.59)	519 (34.81)	0.072
Peptic ulcer, n (%)	20 (3.58)	43 (2.88)	0.418
Previous stroke, n (%)	35 (6.26)	130 (8.72)	0.069
Previous Bleeding, n (%)	6 (1.07)	23 (1.54)	0.423
Previous MI, n (%)	9 (1.61)	27 (1.81)	0.758
AF type			
Paroxysmal, n (%)	275 (49.19)	710 (47.62)	0.525
Persistent, n (%)	183 (32.74)	455 (30.52)	0.334
Permanent, n (%)	101 (18.07)	326 (21.86)	0.059
CHA2DS2-VASc score	3.14 ± 1.03	3.65 ± 1.22	0.422
HAS-BLED score	2.19 ± 1.38	2.12 ± 1.11	0.696
PCI, n (%)	42 (7.51)	166 (11.13)	0.016*
CABG, n (%)	3 (0.54)	18 (1.21)	0.179
ACEI/ARB, n (%)	357 (63.86)	993 (66.60)	0.245
β-blocker, n (%)	374 (66.91)	1041 (69.82)	0.204
Statins, n (%)	495 (88.55)	1288 (86.38)	0.194
Diuretics, n (%)	81 (14.49)	207 (13.88)	0.725
Digoxin, n (%)	91 (16.28)	287 (19.25)	0.123
CCB, n (%)	132 (23.61)	318 (21.33)	0.266
Proton pump inhibitors, n (%)	315 (56.35)	863 (57.88)	0.533
INR	2.16 ± 0.42	2.27 ± 0.38	0.236

Data are expressed as the mean ± standard deviation or number (%) of subjects. *Statically significant at *p* < 0.05. Abbreviations: *SCAD* stable coronary artery disease, *ACS* acute coronary syndrome, *AF* atrial fibrillation, *MI* myocardial infarction, *PCI* percutaneous coronary intervention, *CABG* coronary artery bypass graft, *ACEI* angiotensin-converting enzyme inhibitor, *ARB* angiotensin receptor blocker, *CCB* calcium receptor antagonist, *INR* international normalized ratio

### Antithrombotic regimen in patients enrolled each year

**Fig. 2:**
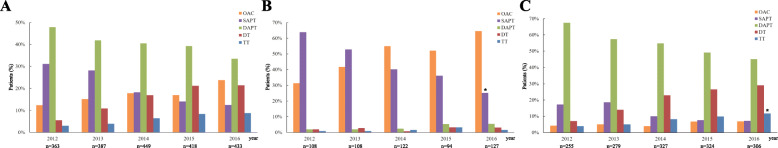
Antithrombotic therapy in newly enrolled patients each year. The antithrombotic regimen trends in (**a**) the total population, (**b**) SCAD+AF patients and (**c**) ACS + AF patients included from 2012 to 2016. *Statically significant for trend at *p* < 0.05. Abbreviations: SCAD: stable coronary artery disease; ACS: acute coronary syndrome; AF: atrial fibrillation; OAC: oral anticoagulant; SAPT: single antiplatelet therapy; DAPT: double antiplatelet therapy; DT: dual therapy; TT: triple therapy

As shown in Fig. 2a, we assessed the antithrombotic regimen in patients with CAD and AF at discharge and annually thereafter. The most common drug prescribed was DAPT. Although the proportion of DAPT continuously decreased (47.93, 41.86, 40.53, 39.23 and 33.49%, respectively) and the use of DT (5.51, 10.85, 16.93, 21.19 and 21.48%, respectively) and TT (3.03, 3.88, 6.46, 8.37 and 8.80%, respectively) gradually increased over the years, the changes were not statistically significant (*p* = 0.055, *p* = 0.053 and *p* = 0.051 for trend, respectively). In the SCAD+AF group, the proportion of patients receiving SAPT clearly decreased over the study period (63.89, 52.78, 40.16, 36.17 and 25.20%, respectively, *p* = 0.048 for trend), and OAC monotherapy was the most common treatment beginning in 2014 (Fig. 2b). Figure 2c shows that the proportion of patients prescribed TT was small and steadily increased each (3.92, 5.02, 8.26, 9.88 and 11.76%, respectively, *p* = 0.048 for trend) in the ACS + AF group. In addition, the rate of DAPT use tended to decrease (67.45, 57.35, 54.74, 49.07 and 45.10%, respectively) and that of DT used showed an increasing tendency (7.06, 13.98, 22.94, 26.54 and 29.08%, respectively) from 2012 to 2016, but no significant difference was observed (*p* = 0.050 and *p* = 0.051 for trend, respectively).

### Antithrombotic strategy at discharge and during follow-up

**Fig. 3:**
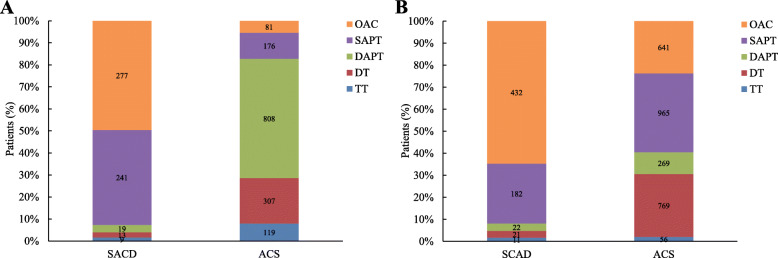
Antithrombotic strategy at discharge and at annual follow-up. Choice of antithrombotic therapy in patients in the SCAD+AF and ACS + AF groups (**a**) at discharge and (**b**) in the follow-up period. Abbreviations: SCAD: stable coronary artery disease; ACS: acute coronary syndrome; AF: atrial fibrillation; OAC: oral anticoagulant; SAPT: single antiplatelet therapy; DAPT: double antiplatelet therapy; DT: dual therapy; TT: triple therapy

Figure 3a shows that OAC monotherapy and SAPT were the most common antithrombotic therapies, and the proportions of the two therapies were similar (49.55 and 43.11%, respectively) in the SCAD+AF group at discharge, while most patients in the ACS + AF group received DAPT and DT (54.19 and 20.59%, respectively). During follow-up, we recorded the change in antithrombotic treatments compared with previous treatments (Fig. 3b). In the SCAD+AF group, OAC monotherapy was still the most frequently used therapy, and the proportion of OAC monotherapy was higher than that of SAPT (64.67 and 27.25%, respectively). The proportion of OAC significantly increased (64.67% vs. 49.55%, *p* < 0.001), while the proportion of SAPT significantly decreased (27.25% vs. 43.11%, *p* < 0.001) during follow-up compared to the same parameters at discharge. In the ACS + AF group, SAPT and DT were the most common treatments among patients (35.74 and 28.48%, respectively), and the proportion of patients using DAPT significantly decreased (9.96% vs. 54.19%, *p* < 0.001), while the proportions of patients using SAPT and DT significantly increased (35.74% vs. 11.80%, *p* < 0.001 and 28.48% vs. 20.59%, *p* < 0.001, respectively) during follow-up when compared to the same parameters at discharge.

### Clinical complications and the corresponding antithrombotic strategy during follow-up

The incidence of adverse clinical outcomes in the SCAD+AF group and ACS + AF group during the follow-up of 34 ± 13 months is indicated in Table 2, including 23 patients (1.12%) with ischaemic stroke, 93 patients (4.54%) with bleeding, 19 patients (0.93%) with MI and 39 patients (1.90%) experiencing all-cause death events. The ACS + AF group had a higher incidence of bleeding and all-cause death events than the SCAD+AF group (5.37% vs. 2.33%, *p* = 0.003 and 2.28% vs. 0.89%, *p* = 0.041, respectively). The incidence of mild and moderate bleeding events was higher in the ACS + AF group than in the SCAD+AF group (3.35% vs. 1.61%, *p* = 0.035 and 1.74% vs. 0.54%, *p* = 0.039, respectively). There was no significant difference in major bleeding, ischaemic stroke or MI event rates between the 2 groups (0.27% vs. 0.18%, *p* = 1.000; 1.07% vs. 1.17%, *p* = 0.732 and 1.01% vs. 0.72%, *p* = 0.725, respectively). Compared to warfarin monotherapy, the use of non-vitamin K antagonist (VKA) oral anticoagulant (NOAC) alone significantly decreased the incidences of thromboembolism and bleeding events in patients with CAD and AF (1.91% vs. 6.97%, *p* = 0.048 and 1.27% vs. 5.91%, *p* = 0.046, respectively, Supplement Table 2). In addition, NOAC was associated with a lower incidence of bleeding events than warfarin in patients prescribed OAC-SAPT combined therapy (2.68% vs. 9.62%, *p* = 0.039).

**Table 2:** The incidence of major adverse outcomes during follow-up

	n (%)	OAC	SAPT	DAPT	DT	TT	***P***-value
**Ischemic stroke**	23 (1.12)	4 (0.20)	12 (0.59)	6 (0.29)	1 (0.05)	0 (0)	
SCAD+AF	7 (1.25)	1 (0.18)	4 (0.72)	2 (0.36)	0 (0)	0 (0)	
ACS + AF	16 (1.07)	3 (0.20)	8 (0.54)	4 (0.27)	1 (0.07)	0 (0)	0.732
**Bleeding**	93 (4.54)	14 (0.68)	7 (0.34)	20 (0.05)	23 (1.12)	29 (1.41)	
SCAD+AF	13 (2.33)	5 (0.89)	2 (0.36)	1 (0.18)	1 (0.18)	4 (0.72)	
ACS + AF	80 (5.37)	9 (0.60)	5 (0.34)	19 (1.27)	22 (1.48)	25 (1.68)	0.003*
**mild bleeding**	59 (2.88)	11 (0.54)	6 (0.29)	12 (0.59)	16 (0.78)	14 (0.68)	
SCAD+AF	9 (1.61)	4 (0.72)	2 (0.36)	0 (0)	1 (0.18)	2 (0.36)	
ACS + AF	50 (3.35)	7 (0.47)	4 (0.27)	12 (0.80)	15 (1.01)	12 (0.80)	0.035*
**moderate bleeding**	29 (1.41)	3 (0.15)	1 (0.05)	7 (0.34)	6 (0.29)	12 (0.59)	
SCAD+AF	3 (0.54)	1 (0.18)	0 (0)	1 (0.18)	0 (0)	1 (0.18)	
ACS + AF	26 (1.74)	2 (0.13)	1 (0.07)	6 (0.40)	6 (0.40)	11 (0.74)	0.039*
**major bleeding**	5 (0.24)	0 (0)	0 (0)	1 (0.05)	1 (0.05)	3 (0.15)	
SCAD+AF	1 (0.18)	0 (0)	0 (0)	0 (0)	0 (0)	1 (0.18)	
ACS + AF	4 (0.27)	0 (0)	0 (0)	1 (0.07)	1 (0.07)	2 (0.13)	1.000
**MI**	19 (0.93)	7 (0.34)	5 (0.24)	2 (0.10)	3 (0.15)	2 (0.10)	
SCAD+AF	4 (0.72)	2 (0.36)	2 (0.36)	0 (0)	0 (0)	0 (0)	
ACS + AF	15 (1.01)	5 (0.34)	3 (0.20)	2 (0.13)	3 (0.20)	2 (0.13)	0.725
**All-cause death**	39 (1.90)	11 (0.54)	8 (0.39)	7 (0.34)	4 (0.20)	9 (0.44)	
SCAD+AF	5 (0.89)	1 (0.18)	2 (0.36)	0 (0.00)	1 (0.18)	1 (0.18)	
ACS + AF	34 (2.28)	10 (0.67)	6 (0.40)	7 (0.47)	3 (0.20)	8 (0.54)	0.041*

*Statically significant at *p* < 0.05. Abbreviations: *SCAD* stable coronary artery disease, *ACS* acute coronary syndrome, *AF* atrial fibrillation, *MI* myocardial infarction, *OAC* oral anticoagulant, *SAPT* single antiplatelet therapy, *DAPT* double antiplatelet therapy, *DT* dual therapy, *TT* triple therapy

### Independent predictive factors of adverse outcomes

According to multivariate Cox regression analysis, age, hypertension and prior stroke were independently associated with an increased risk of ischaemic stroke in the SCAD+AF group (odds ratio [OR], 1.03, 95% confidence interval [CI], 1.01–1.06, *p* = 0.007; OR, 1.33, 95% CI, 1.04–1.72, *p* = 0.014 and OR, 1.39, 95% CI, 1.12–2.17, *p* = 0.009, respectively; Table 3) and the ACS + AF group (OR, 1.44, 95% CI, 1.06–1.98, *p* = 0.006; OR, 1.50, 95% CI, 1.22–1.83, *p* = 0.020 and OR, 1.25, 95% CI, 1.04–1.56, *p* = 0.031, respectively). OAC was independently associated with a decreased risk of ischaemic stroke in both groups (OR, 0.33, 95% CI, 0.25–0.46, *p* = 0.016 and OR, 0.54, 95% CI, 0.30–0.93, *p* = 0.005, respectively). Age and previous bleeding independently increased the risk of bleeding in the ACS + AF group (OR, 1.85, 95% CI, 1.28–2.66, *p* = 0.020 and OR, 1.78, 95% CI, 1.33–2.39, *p* = 0.006, respectively), while only a history of bleeding independently increased the risk of haemorrhage in the SCAD+AF group (OR, 1.85, 95% CI, 1.12–3.07, *p* = 0.001). Multivariate Cox regression analysis also revealed that APT was an independent protective factor for MI in the SCAD+AF group (OR, 0.71, 95% CI, 0.58–0.86, *p* = 0.004) and the ACS + AF group (OR, 0.66, 95% CI, 0.56–0.79, *p* < 0.001), and diabetes mellitus was an independent risk factor for MI in the ACS + AF group (OR, 2.23; 95% CI, 1.75–2.83, *p* = 0.001). Age and heart failure were independently associated with an increased risk of all-cause death in the SCAD+AF group (OR, 1.67; 95% CI, 1.37–2.02, *p* = 0.005 and OR, 2.81; 95% CI, 1.82–4.34, *p* = 0.028, respectively) and the ACS + AF group (OR, 1.47; 95% CI, 1.21–1.79, *p* = 0.001 and OR, 3.02; 95% CI, 1.61–5.68, *p* = 0.003, respectively). OAC (OR, 0.32; 95% CI, 0.18–0.57, *p* < 0.001 and OR, 0.47; 95% CI, 0.28–0.77, *p* = 0.009, respectively) and APT (OR, 0.71; 95% CI, 0.54–0.95, *p* = 0.015 and OR, 0.57; 95% CI, 0.42–0.75, *p* < 0.001, respectively) were independently associated with a decreased risk of all-cause death in both groups.

**Table 3:** Independent predictors of major adverse outcomes by multivariable Cox regression analysis in SCAD+AF group and ACS + AF group

		SCAD + AF		ACS + AF	
		OR (95% CI)	***P***-value	OR (95% CI)	***P***-value
**Ischemic stroke**	Age ≥ 65	1.03 (1.01–1.06)	0.007*	1.44 (1.06–1.98)	0.006*
	Female	1.35 (0.85–1.99)	0.179	1.21 (0.93–1.60)	0.117
	Hypertension	1.33 (1.04–1.72)	0.014*	1.50 (1.22–1.83)	0.020*
	Heart failure	0.99 (0.94–1.03)	0.225	1.26 (0.89–1.78)	0.193
	Diabetes mellitus	0.62 (0.27–1.46)	0.214	0.92 (0.36–2.27)	0.890
	Previous stroke	1.39 (1.12–2.17)	0.009*	1.25 (1.04–1.56)	0.031*
	Previous bleeding	1.68 (0.77–3.68)	0.180	1.16 (0.57–2.42)	0.713
	Coronary stent	0.81 (0.31–2.26)	0.700	0.78 (0.37–1.53)	0.440
	OAC	0.33 (0.25–0.46)	0.016*	0.54 (0.30–0.93)	0.005*
	APT	0.72 (0.42–1.10)	0.113	0.88 (0.44–1.65)	0.637
**Bleeding**	Age ≥ 65	1.29 (0.51–3.35)	0.587	1.85 (1.28–2.66)	0.020*
	Female	1.30 (0.57–2.93)	0.508	0.90 (0.76–1.04)	0.162
	Hypertension	1.10 (0.74–1.63)	0.482	1.14 (0.95–1.33)	0.148
	Heart failure	1.04 (0.75–1.45)	0.912	0.67 (0.39–1.13)	0.134
	Diabetes mellitus	0.95 (0.77–1.16)	0.872	1.05 (0.87–1.23)	0.960
	Previous stroke	1.17 (0.42–3.27)	0.353	1.55 (0.70–3.45)	0.172
	Previous bleeding	1.85 (1.12–3.07)	0.001*	1.78 (1.33–2.39)	0.006*
	Coronary stent	0.67 (0.41–1.11)	0.621	1.34 (0.67–2.70)	0.316
	OAC	1.17 (0.56–2.25)	0.658	2.28 (0.58–3.91)	0.243
	APT	1.32 (0.86–2.01)	0.137	2.18 (0.71–6.73)	0.171
**MI**	Age ≥ 65	1.65 (0.73–3.74)	0.240	1.16 (0.56–2.13)	0.723
	Female	1.55 (0.83–2.91)	0.133	0.93 (0.59–1.46)	0.738
	Hypertension	1.34 (0.82–2.17)	0.224	2.29 (0.71–7.40)	0.166
	Heart failure	0.79 (0.40–1.62)	0.557	0.99 (0.91–2.08)	0.787
	Diabetes mellitus	1.26 (0.60–3.31)	0.453	2.23 (1.75–2.83)	0.001*
	Previous stroke	1.42 (0.82–2.60)	0.178	1.71 (0.69–4.27)	0.248
	Previous bleeding	1.28 (0.70–2.64)	0.430	1.51 (0.96–2.37)	0.308
	Coronary stent	1.03 (0.89–1.17)	0.713	0.95 (0.81–1.10)	0.558
	OAC	0.90 (0.57–1.38)	0.628	1.30 (0.65–2.58)	0.639
	APT	0.71 (0.58–0.86)	0.004*	0.66 (0.56–0.79)	< 0.001*
**All-cause death**	Age ≥ 65	1.67 (1.37–2.02)	0.005*	1.47 (1.21–1.79)	0.001*
	Female	0.85 (0.57–1.28)	0.470	0.93 (0.66–1.37)	0.750
	Hypertension	1.01 (0.96–1.04)	0.615	1.12 (0.93–1.31)	0.087
	Heart failure	2.81 (1.82–4.34)	0.028*	3.02 (1.61–5.68)	0.003*
	Diabetes mellitus	0.84 (0.52–1.19)	0.390	0.96 (0.67–1.38)	0.839
	Previous stroke	1.11 (0.87–1.38)	0.380	1.40 (0.55–3.23)	0.560
	Previous bleeding	2.07 (0.72–5.71)	0.184	1.28 (0.85–1.91)	0.215
	Coronary stent	0.76 (0.54–1.09)	0.119	0.70 (0.42–1.17)	0.148
	OAC	0.32 (0.18–0.57)	< 0.001*	0.47 (0.28–0.77)	0.009*
	APT	0.71 (0.54–0.95)	0.015*	0.57 (0.42–0.75)	< 0.001*

*Statically significant at *p* < 0.05. Abbreviations: *OR* odds ratio, *CI* confidence interval, *SCAD* stable coronary artery disease, *ACS* acute coronary syndrome, *AF* atrial fibrillation, *MI* myocardial infarction, *OAC* oral anticoagulant, *APT* antiplatelet therapy

## Discussion

The major findings of the current study were as follows: (1) OAC was the most frequently implemented treatment prescribed at discharge in SCAD+AF patients, whereas DAPT was the most common treatment in ACS + AF patients; (2) During the follow-up, the proportions of patients using OAC and SAPT significantly increased and decreased, respectively, in the SCAD+AF group, and the use of DAPT significantly decreased while the proportions of patients using SAPT and DT significantly increased in the ACS + AF group when compared to the use of these treatments at discharge; (3) Compared to the SCAD+AF group, the incidence of bleeding and all-cause death events was significantly higher in the ACS + AF group; (4) Multivariate analysis showed that OAC was an independent protective factor for ischaemic stroke, and previous bleeding was independently associated with an increased risk of bleeding in both groups; and (5) NOAC was superior to warfarin in patients with CAD and AF.

Of particular concern in CAD management has been the use of APT in patients with AF receiving OAC for stroke prevention when their CHA_2_DS_2_-VASc score is ≥2. Aspirin is still the gold standard for secondary prevention in SCAD patients, with clopidogrel being an alternative treatment, and aspirin has been indicated to decrease major cardiovascular events by approximately 20 to 25% [15, 16]. Therefore, patients with SCAD and AF may, in theory, need treatment with DT to avoid thromboembolic and recurrent cardiovascular events [17]. Consistent with our findings that a higher incidence of bleeding events occurred in patients using DT or TT than in those using SAPT or OAC monotherapy in the SACD+AF group, registry studies showed that the risk of haemorrhage increased 2-fold when APT and warfarin were used simultaneously [18]. The same result was also found among patients taking a combination of NOAC and APT [19, 20]. Contemporary guidelines and expert consensus built on observational data [5, 6, 21] suggested using OAC alone as the default strategy to reduce haemorrhage events in such patients [3, 22]. In our study, the most common treatment beginning in 2014 was OAC monotherapy in the SCAD+AF patients newly enrolled each year, and prescription of OAC alone was the most commonly used treatment in SCAD patients with AF at discharge and during follow-up. The proportions of patients prescribed OAC monotherapy and SAPT significantly increased and decreased, respectively, in the follow-up period when compared to the proportions at discharge in the SCAD+AF group, which supported the above viewpoint. A multicentre, open-label trial carried out in Japan also showed that rivaroxaban (a NOAC) monotherapy was not inferior to the combined treatment (rivaroxaban plus a single antiplatelet agent) in regard to efficacy and was superior in regard to safety in patients with SCAD and AF [9]. The rate of DT use was also low in patients in the SCAD+AF group. Indeed, most observational studies indicated that DT was linked with a higher risk of haemorrhage without exerting obvious positive effects on thromboembolic outcomes [5, 6, 21]. Nevertheless, concomitant antiplatelet therapy was essential for patients with SCAD and AF in the case of coronary revascularization, after which DAPT should be recommended for 6 to 12 months in most patients except those at a high risk of bleeding, for whom 1–3 months of DAPT may be more acceptable [8, 23, 24]. A small number of SCAD+AF patients were found to choose DAPT and anticoagulant-antiplatelet combined therapy in this study, which may be due to PCI. Moreover, as the 2018 European Heart Rhythm Association recommended, changing from DAPT to NOAC alone early (e.g., at 6 months) could be an alternative for SCAD+AF patients at low ischaemic and high haemorrhagic risks after PCI [9].

Patients with ACS and AF need to be treated with OAC for protection against ischaemic stroke and with DAPT for the prevention of ischaemic events such as stent thrombosis and MI. In the European Society of Cardiology (ESC) guidelines for ACS patients with AF, TT was still recommended as the default strategy [3]. Although our data showed a steadily increasing proportion of TT use in ACS + AF patients from 2012 to 2016, DAPT was the most frequently prescribed treatment rather than TT at discharge, and this relatively conservative choice may be due to the higher risk of bleeding events associated with TT [18, 19, 25]. In this study, we also demonstrated that NOAC alone significantly decreased the incidences of thromboembolism and bleeding events in patients with CAD and AF compared to the effects of warfarin monotherapy. Two methods that are available for reducing this high bleeding risk are discontinuing aspirin and using NOAC instead of VKAs due to their better safety profile [26]. Three trials, including WOEST (*n* = 573,25% with ACS) [11], PIONEER AF-PCI (*n* = 2124, 52% with ACS) [27] and RE-DUAL PCI (*n* = 2725,51% with ACS) [28], have investigated the concept of discontinuing aspirin after PCI and thus using DT to treat these patients. Compared to the TT group, major bleeding significantly decreased in the DT groups (NOAC plus P2Y12 inhibition alone) of all three trials. We also found that the proportion of ACS patients with AF being treated with DAPT and DT in the follow-up period exhibited a decreasing and increasing trend, respectively, when compared to the proportions at discharge. In addition, among the newly included population with ACS and AF each year, the number of people using DT at discharge showed an increasing tendency despite the fact that no significant difference was observed. Our study suggested that a relatively low number of ACS + AF patients received PCI because over 70% of patients were diagnosed with unstable angina, and the majority of these patients had serious coronary artery lesions (< 70%). Patients preferred to choose short-term drug treatment if it could alleviate the symptoms of the disease. Nevertheless, the ACS + AF group had a higher incidence of bleeding than the SCAD+AF group, which may be because more patients underwent PCI and were treated with DAPT, DT and TT [29].

The results of this study indicated the current status of low anticoagulant therapy in patients with CAD and AF. This finding was consistent with the China Registry of Atrial Fibrillation (CRAF), in which 25.6% of patients with nonvalvular atrial fibrillation (NVAF) and a CHA2DS2VASc score ≥ 2 received oral anticoagulant monotherapy or anticoagulant-antiplatelet combined therapy [30]. The clinical characteristics of NVAF patients in China and the higher risk of intracranial haemorrhage with anticoagulant treatment in Chinese patients than in patients of other racial groups may affect the utilization of anticoagulation therapy in clinical practice [31]. The results also indicated that NOAC was superior to warfarin in patients with CAD and AF. The multivariable Cox regression model showed that OAC was an independent protective factor against ischaemic stroke in both groups. These results indirectly reminded us of the urgent need to improve the guidelines on antithrombotic drug selection and management and to support the application of NOAC, especially for patients who undergo coronary revascularization and are at high risk of haemorrhage. Thus, when physicians consider TT as antithrombotic therapy in patients with ACS and AF, NOAC combined with P2Y12 platelet inhibitor may be a better choice. However, the related research is not conclusive. The multivariable Cox regression analysis also suggested that heart failure was significantly associated with all-cause death, and it had been reported that the progression of heart failure after ACS led to an especially poor overall prognosis [32, 33], which may explain the higher mortality in the ACS + AF group in this study.

## Conclusions

This study suggested that the prescription rate of anticoagulant-antiplatelet combined therapy was low in ACS + AF patients with a high risk of stroke. In clinical practice, the awareness of anticoagulation needs to be strengthened in regard to patients with CAD and AF. The optimal antithrombotic regimen in patients with CAD and AF remains unclear when the CHA_2_DS_2_-VASc score is ≥2. Further adequately powered randomized controlled trials (RCTs) are needed to guide clinical decisions.

### Limitations

Our study has several limitations. First, the study has a small sample size. Second, this study is a single-centre, observational retrospective study and may not adequately represent the current status of antithrombotic therapy in the whole area. Third, because the follow-up was carried out over the telephone, it is difficult for us to assess the INR of patients in a timely manner when patients adjust medications or when adverse events occur, which led to a possible risk of bias. Fourth, there are insufficient data about the exact dose of antiplatelet and anticoagulant drugs, which may affect the analysis of adverse outcomes [34]. In addition, we did not compare the effects of the same type of antithrombotic drugs on patients [16, 35]. Finally, NOAC did not enter the market until 2013 in China, and these drugs cannot be reimbursed by medical insurance. Therefore, the number of patients prescribed NOAC in this study was low. However, this analysis provides information on the current situation and time trends in the antithrombotic strategy, and the relationship between these incomplete data and clinical endpoints deserves to be further analysed.

## Supplementary information

**Additional file 1: Supplement Table 1.** Warfarin duration and Levels of INR in patients at discharge.

**Additional file 2: Supplement Table 2.** The incidences of thromboembolism and bleeding events in patients prescribed warfarin or NOAC during follow-up.

## Data Availability

The datasets that support the findings of this study are available from the corresponding author on reasonable request.

## Abbreviations

APT: Antiplatelet therapy
OAC: Oral anticoagulant
SCAD: Stable coronary heart disease
ACS: Acute coronary syndromes
AF: Atrial fibrillation
CAD: Coronary artery disease
MI: Myocardial infarction
TT: Triple therapy
DAPT: Double antiplatelet therapy
ECG: Electrocardiogram
CAG: Coronary arteriography
CTA: Computed tomography angiography
INR: International normalized ratio
STEMI: ST-segment elevation myocardial infarction
NSTEMI: Non- ST-segment elevation myocardial infarction
SAPT: Single antiplatelet therapy
DT: Dual therapy
PCI: Percutaneous coronary intervention
OR: Odds ratio
CI: Confidence interval
NOAC: Non-VKA oral anticoagulant
ESC: European society of cardiology
GUSTO: Global utilization of streptokinase and tissue plasminogen activator for occluded coronary arteries
CRAF: China registry of atrial fibrillation
NVAF: Nonvalvular atrial fibrillation
