# Supplementary material for: Antithrombotic therapy in coronary artery disease patients with atrial fibrillation
Source: BMC Cardiovasc Disord. 2020 Jul 6;20:323. doi: 10.1186/s12872-020-01609-8 (PMC7339421; doi:10.1186/s12872-020-01609-8)
Supplement: Supplementary file 1 — Additional file 1: Supplement Table 1. Warfarin duration and Levels of INR in patients at discharge. [file 12872_2020_1609_MOESM1_ESM.docx]

**Supplement Table 1.** **Warfarin duration and Levels of INR in patients at discharge**

|  | **Warfarin monotherapy (n=201)** | **Warfarin + SAPT (n=208)** | **Warfarin + DAPT (n=128)** |
| --- | --- | --- | --- |
| **SCAD+AF group, n (%)**  **< 1 month, n (%)** | 156 (77.61)  29 (14.43) | 10 (4.81)  2 (0.96) | 9 (7.03)  3 (2.34) |
| INR < 2, n (%) | 17 (8.46) | 2 (0.96) | 2 (1.56) |
| INR 2.0-3.0, n (%) | 12 (5.97) | 0 (0) | 1 (0.78) |
| INR > 3, n (%) | 0 (0) | 0 (0) | 0 (0) |
| **1 month, n (%)** | 18 (8.96) | 0 (0) | 1 (0.78) |
| INR < 2, n (%) | 1 (0.50) | 0 (0) | 1 (0.78) |
| INR 2.0-3.0, n (%) | 17 (8.46) | 0 (0) | 0 (0) |
| INR > 3, n (%)  **3 months, n (%)**  INR < 2, n (%)  INR 2.0-3.0, n (%)  INR > 3, n (%)  **6 months, n (%)**  INR < 2, n (%)  INR 2.0-3.0, n (%)  INR > 3, n (%)  **12 months, n (%)**  INR < 2, n (%)  INR 2.0-3.0, n (%)  INR > 3, n (%)  **> 1 year, n (%)**  INR < 2, n (%)  INR 2.0-3.0, n (%)  INR > 3, n (%)  **ACS+AF group**  **< 1 month, n (%)**  INR < 2, n (%)  INR 2.0-3.0, n (%)  INR > 3, n (%)  **1 month, n (%)**  INR < 2, n (%)  INR 2.0-3.0, n (%)  INR > 3, n (%)  **3 months, n (%)**  INR < 2, n (%)  INR 2.0-3.0, n (%)  INR > 3, n (%)  **6 months, n (%)**  INR < 2, n (%)  INR 2.0-3.0, n (%)  INR > 3, n (%)  **12 months, n (%)**  INR < 2, n (%)  INR 2.0-3.0, n (%)  INR > 3, n (%)  **> 1 year, n (%)**  INR < 2, n (%)  INR 2.0-3.0, n (%)  INR > 3, n (%) | 0 (0)  21 (10.45)  1 (0.50)  20 (9.95)  0 (0)  25 (12.44)  0 (0)  25 (12.44)  0 (0)  24 (11.94)  0 (0)  24 (11.94)  0 (0)  39 (19.40)  0 (0)  39 (19.40)  0 (0)  45 (22.39)  2 (1.00)  0 (0)  2 (1.00)  0 (0)  7 (3.48)  1 (0.50)  6 (2.99)  0 (0)  10 (4.98)  0 (0)  10 (4.98)  0 (0)  13 (6.47)  1 (0.50)  12 (5.97)  0 (0)  7 (3.48)  0 (0)  7 (3.48)  0 (0)  6 (2.99)  0 (0)  6 (2.99)  0 (0) | 0 (0)  1 (0.48)  0 (0)  1 (0.48)  0 (0)  2 (0.96)  0 (0)  2 (0.96)  0 (0)  2 (0.96)  0 (0)  2 (0.96)  0 (0)  3 (1.44)  0 (0)  3 (1.44)  0 (0)  198 (95.19)  13 (6.25)  6 (2.88)  7 (3.37)  0 (0)  23 (11.06)  1 (0.48)  22 (10.58)  0 (0)  29 (14.43)  1 (0.48)  28 (13.46)  0 (0)  47 (22.60)  0 (0)  47 (22.60)  0 (0)  45 (21.63)  0 (0)  45 (21.63)  0 (0)  41 (19.71)  0 (0)  41 (19.71)  0 (0) | 0 (0)  1 (0.78)  0 (0)  1 (0.78)  0 (0)  2 (1.56)  0 (0)  2 (1.56)  0 (0)  1 (0.78)  0 (0)  1 (0.78)  0 (0)  1 (0.78)  0 (0)  1 (0.78)  0 (0)  119 (92.97)  11 (8.59)  7 (5.47)  4 (3.12)  0 (0)  15 (11.72)  1 (0.78)  14 (10.94)  0 (0)  22 (17.19)  1 (0.78)  21 (16.41)  0 (0)  21 (16.41)  0 (0)  21 (16.41)  0 (0)  25 (19.53)  0 (0)  25 (19.53)  0 (0)  25 (19.53)  0 (0)  25 (19.53)  0 (0) |

Abbreviations: SCAD: stable coronary artery disease; ACS: acute coronary syndrome; AF: atrial fibrillation; SAPT: single antiplatelet therapy; DAPT: double antiplatelet therapy; INR, international normalized ratio.
