# Supplementary material for: Antithrombotic therapy in coronary artery disease patients with atrial fibrillation
Source: BMC Cardiovasc Disord. 2020 Jul 6;20:323. doi: 10.1186/s12872-020-01609-8 (PMC7339421; doi:10.1186/s12872-020-01609-8)
Supplement: Supplementary file 2 — Additional file 2: Supplement Table 2. The incidences of thromboembolism and bleeding events in patients prescribed warfarin or NOAC during follow-up. [file 12872_2020_1609_MOESM2_ESM.docx]

**Supplement Table 2. The incidences of thromboembolism and bleeding events in patients prescribed warfarin or NOAC during follow-up**

|  | **OAC monotherapy (n=358)** | | |  | **DT (n=320)** | | | |
| --- | --- | --- | --- | --- | --- | --- | --- | --- |
|  | **Warfarin**  **(n=201)** | **NOAC**  **(n=157)** | ***P*-value** |  | **Warfarin + SAPT**  **(n=208)** | **NOAC + SAPT**  **(n=112)** | ***P*-value** | |
| **Thromboembolism n (%)** | 14 (6.97) | 3 (1.91) | 0.048* |  | 6 (2.88) | 1 (0.89) | | 0.447 |
| **Bleeding n (%)** | 12 (5.97) | 2 (1.27) | 0.046* |  | 20 (9.62) | 3 (2.68) | | 0.039* |

*Statically significant at p<0.05. Abbreviations: OAC: oral anticoagulant; NOAC: non-VKA oral anticoagulant; DT: dual therapy; SAPT: single antiplatelet therapy.
